# Brain tissue- and cell type-specific eQTL Mendelian randomization reveals efficacy of *FADS1* and *FADS2* on cognitive function

**DOI:** 10.1038/s41398-024-02784-4

**Published:** 2024-02-05

**Authors:** Xueyan Wu, Lei Jiang, Hongyan Qi, Chunyan Hu, Xiaojing Jia, Hong Lin, Shuangyuan Wang, Lin Lin, Yifang Zhang, Ruizhi Zheng, Mian Li, Tiange Wang, Zhiyun Zhao, Min Xu, Yu Xu, Yuhong Chen, Jie Zheng, Yufang Bi, Jieli Lu

**Affiliations:** 1 Department of Endocrine and Metabolic Diseases, Shanghai Institute of Endocrine and Metabolic Diseases, Ruijin Hospital, Shanghai Jiao Tong University School of Medicine, Shanghai, China; 2 Shanghai National Clinical Research Center for Endocrine and Metabolic Diseases, Key Laboratory for Endocrine and Metabolic Diseases of the National Health Commission of the PR China, Shanghai National Center for Translational Medicine, Ruijin Hospital, Shanghai Jiao Tong University School of Medicine, Shanghai, China; 3 Network and Information Center, Shanghai Jiao Tong University, Shanghai, China; 4 Shanghai Digital Medicine Innovation Center, Ruijin Hospital, Shanghai Jiao Tong University School of Medicine, Shanghai, China; 5 MRC Integrative Epidemiology Unit (IEU), Bristol Medical School, University of Bristol, Oakfield House, Oakfield Grove, Bristol, BS8 2BN UK

**Keywords:** Medical genetics, Drug discovery

## Abstract

Epidemiological studies suggested an association between omega-3 fatty acids and cognitive function. However, the causal role of the fatty acid desaturase (*FADS*) gene, which play a key role in regulating omega-3 fatty acids biosynthesis, on cognitive function is unclear. Hence, we used two-sample Mendelian randomization (MR) to estimate the gene-specific causal effect of omega-3 fatty acids (*N* = 114,999) on cognitive function (*N* = 300,486). Tissue- and cell type-specific effects of *FADS1*/*FADS2* expression on cognitive function were estimated using brain tissue cis-expression quantitative trait loci (cis-eQTL) datasets (GTEx, *N* ≤ 209; MetaBrain, *N* ≤ 8,613) and single cell cis-eQTL data (*N* = 373), respectively. These causal effects were further evaluated in whole blood cis-eQTL data (*N* ≤ 31,684). A series of sensitivity analyses were conducted to validate MR assumptions. Leave-one-out MR showed a *FADS* gene-specific effect of omega-3 fatty acids on cognitive function [β = −1.3 × 10^−2^, 95% confidence interval (CI) (−2.2 × 10^−2^, −5 × 10^−3^), *P* = 2 × 10^−3^]. Tissue-specific MR showed an effect of increased *FADS1* expression in cerebellar hemisphere and *FADS2* expression in nucleus accumbens basal ganglia on maintaining cognitive function, while decreased *FADS1* expression in nine brain tissues on maintaining cognitive function [colocalization probability (PP.H4) ranged from 71.7% to 100.0%]. Cell type-specific MR showed decreased *FADS1*/*FADS2* expression in oligodendrocyte was associated with maintaining cognitive function (PP.H4 = 82.3%, respectively). Increased *FADS1*/*FADS2* expression in whole blood showed an effect on cognitive function maintenance (PP.H4 = 86.6% and 88.4%, respectively). This study revealed putative causal effect of *FADS1*/*FADS2* expression in brain tissues and blood on cognitive function. These findings provided evidence to prioritize *FADS* gene as potential target gene for maintenance of cognitive function.

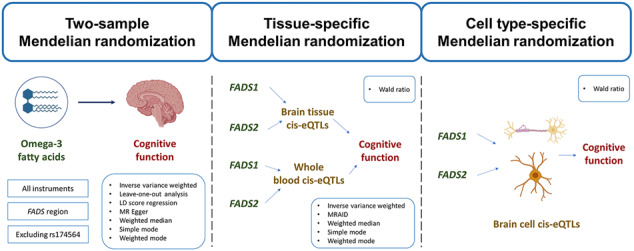

## Introduction

Cognitive dysfunction is an important issue in the aging population [1]. However, changes in brain function start to occur several years before the diagnose of cognitive impairment [2]. Hence, identifying factors associated with the development of cognitive impairment is of great societal interest.

Evidence suggested that fatty acids play an important role in cognition [3]. Previous studies reported that omega-3 fatty acids were associated with cognitive and mental health [4, 5]. However, recent observational studies and randomized controlled trials (RCTs) have shown inconsistent evidence [6–9]. Therefore, other line of evidence is needed to clarify whether there is a causal effect of omega-3 fatty acids on cognitive function. Omega-3 fatty acids were influenced by genetic factors [10–12]. Delta-5 desaturase and delta-6 desaturase are key rate-limiting enzymes that crucial in a series of elongation and desaturation reactions of omega-3 fatty acids, which are encoded by two genes: fatty acid desaturase 1 (*FADS1*) and fatty acid desaturase 2 (*FADS2*) [13, 14]. Several studies reported the associations between single nucleotide polymorphisms (SNPs) in the *FADS* loci and omega-3 fatty acids concentrations [15–17], implying that variants in the *FADS* gene region modify the activity of polyunsaturated fatty acids desaturation. However, evidence between *FADS1*/*FADS2* gene expression and their own cognitive impairment is limited [18]. In addition, *FADS1* and *FADS2* gene are expressed in multiple human tissues and cells. The role of expression levels of *FADS1* and *FADS2* in different tissues and cell types on cognitive function needs further investigation.

Mendelian randomization (MR) analysis is an emerging method that using genetic variants as instrumental variables (IVs) to infer the causal effect of an exposure on an outcome [5, 19–21]. Due to specificity of IVs, the MR estimates are not commonly subject to confounding bias and reverse causation [22]. MR has also been applied to detect putative causal effect of tissue-specific gene expression and a wide range of diseases using expression quantitative trait loci (eQTLs) as instruments [23–25]. However, the eQTL relationship was highly dependent on cell type and eQTLs that from bulk tissue samples may mask the cell specificity of genetic regulatory effects [26]. With development of novel omics tools, especially single-cell sequencing technology [27–29] and genetic colocalization methologies [30], estimating the effect of gene on disease in single-cell level will provide novel insight of disease etiology and molecular mechanism soon. In addition to tissue specificity, recent studies have demonstrated that many eQTL effects are cell type-specific [31], as well as genes showing cell type-specific effects including *FADS1* and *FADS2* [27]. By using eQTLs of diverse cell types will help us to supplement the potential molecular mechanisms that underlie cognitive function.

Therefore, the aim of this study was to investigate the causal effect of omega-3 fatty acids on cognitive function within and outside the *FADS* region by using MR method. To identify potential target gene, the tissue- and cell type-specific causal effects of *FADS1* and *FADS2* gene expression on cognitive function were evaluated using cis-eQTL-based MR and colocalization.

## Methods

### Overall study design

**Fig. 1:**
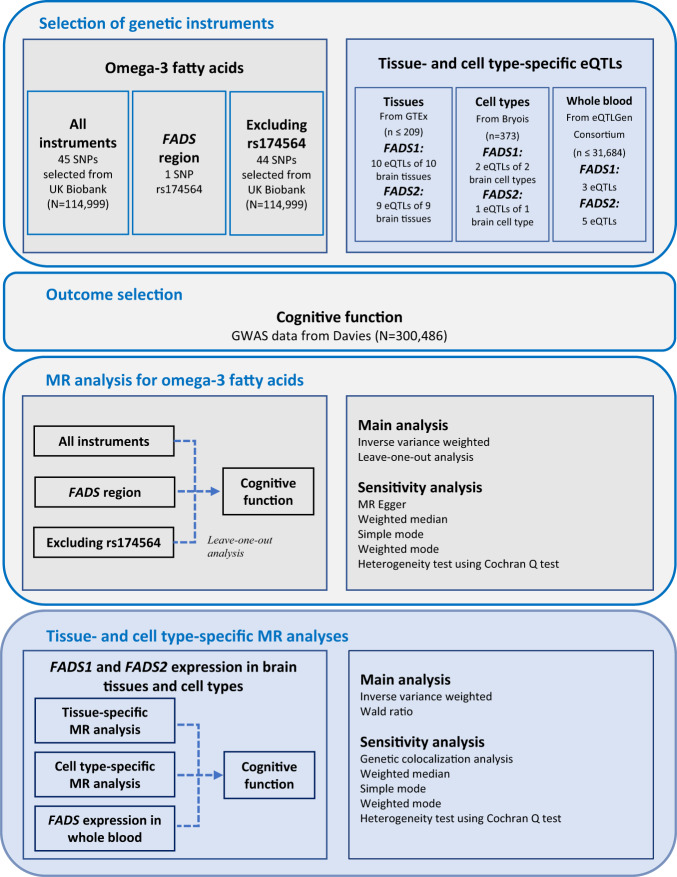
Flow chat of the whole study design. SNP single nucleotide polymorphism, eQTL expression quantitative trait loci, GWAS Genome-wide association study, MR Mendelian randomization.

Figure 1 presented the overall design of the study. In this study, i) we applied a two-sample MR analysis to determine whether omega-3 fatty acids have causal effect on cognitive function within and outside the *FADS* region; ii) conducting tissue- and cell type-specific MR analyses to assess tissue- and cell type-dependent effects of *FADS1* and *FADS2* expression in brain and blood on cognitive function. It is important to note that we applied the MR Steiger filtering approach to exclude cis-eQTLs with potential reverse causality [32]. Ethical approval of all data was obtained in the original studies.

### Data sources

#### Genetic instruments of omega-3 fatty acids

Genome-wide association study (GWAS) results in individuals of mostly European ancestry were obtained from the UK Biobank (up to 114,999 individuals) for plasma concentration of omega-3 fatty acids [33]. This is one of the largest available GWASs of circulating polyunsaturated fatty acids. SNPs were excluded if it had a minor allele frequency no more than 0.01 or did not reach the significant genome-wide association level (*P* ≤ 5 × 10^−8^) (Supplementary Table 1).

#### Genetic instruments of *FADS1* and *FADS2* expression in brain and blood

Brain tissue-specific cis-eQTL data of *FADS1* and *FADS2* expression was obtained from the GTEx project (v8; https://gtexportal.org/home/). For each tissue, the independent cis-eQTL that passed the false discovery rate (FDR) threshold (with FDR < 0.05) was selected as instrument for the tissue-specific analysis, which resulted in 10 cis-eQTLs of the 10 tissues for *FADS1* gene and nine cis-eQTLs of the nine tissues for *FADS2* gene respectively (Supplementary Table 2A). Besides, the results were also validated using the brain cis-eQTL data from the MetaBrain consortium (https://www.metabrain.nl), which is a large scale eQTL meta-analysis of previously published human brain eQTL datasets (*N* ≤ 8,613) [34]. For consistency, we selected the significant cis-eQTLs (q-value < 0.05) for *FADS1* and *FADS2* genes with FDR < 0.05. After selection, three cis-eQTLs of *FADS1* gene derived from three brain tissues were selected (Supplementary Table 2A).

Single-cell cis-eQTL data of *FADS1* and *FADS2* expression was obtained from a brain cell type cis-eQTL study, which including eight brain cell types from 373 brain samples that published by Bryois et al. [27]. The cis-eQTLs (with FDR < 0.05) were identified in two cell types for *FADS1* and one cell type for *FADS2* expression respectively. Same as tissue-specific instruments, only cis-eQTL with the strongest association for each cell type was selected as instrument for the cell-type specific analysis (Supplementary Table 2B).

The cis-eQTL associations of *FADS1* and *FADS2* expression derived from whole blood in 31,470 individuals made available by the eQTLGen Consortium [35], and the study included rigorous quality control (Supplementary Table 1).

#### Outcome data

The GWAS summary statistics of cognitive function was extracted from Davies et al. [3], which included 300,486 individuals of European ancestry from 57 population-based cohorts brought together by the Cohorts for Heart and Aging Research in Genomic Epidemiology (CHARGE), the Cognitive Genomics Consortium (COGENT) consortia, and the UK Biobank. Cognitive function in the three cohorts was estimated by applying a consistent method of extracting a general cognitive function component from cognitive test, which has been reported in more details in the original study [3] (Supplementary Table 3).

### Statistical analyses

#### MR analysis of omega-3 fatty acids on cognitive function

For each omega-3 fatty acids instrument set, we harmonized the SNP-omega-3 fatty acids and SNP-cognitive function data and did the univariable MR analysis by using the TwosampleMR R package (version 0.5.6). In total, 45 SNPs were selected from the UK Biobank as IVs for omega-3 fatty acids, and the primary analysis used the inverse variance weighted (IVW) method to estimate the causal effect.

##### Leave-one-out analysis

We further conducted leave-one-out analysis and assessed the causal effect of single SNP rs174564 within the *FADS* region by using the Wald ratio method [36]. Considering the potential effect of rs174564 on cognitive function, we further excluded it from 45 instruments for omega-3 fatty acids to estimate the causal effect of the other variants outside the *FADS* region on cognitive function.

#### LD Score regression analysis of omega-3 fatty acids on cognitive function

Considering the GWAS data of omega-3 fatty acids and cognitive function have minor sample overlap, which may induce spurious correlation. We employed linkage disequilibrium score regression (LDSC, v.1.0.1) analysis to evaluate the genetic correlation between omega-3 fatty acids and cognitive function and to test the existence of sample overlap [37, 38]. The LD scores from the European 1000 Genomes Project dataset were referenced [39].

#### Tissue- and cell type-specific MR analyses

##### MR analysis of *FADS1* and *FADS2* expression in brain tissues on cognitive function

For tissue-specific MR analysis, we estimated the putative causal effects of *FADS1* expression in 10 brain tissues and *FADS2* expression in nine brain tissues using data from the GTEx. MR analysis of *FADS1* expression in three brain tissues on cognitive function were also conducted using the MetaBrain data. The Wald ratio [36] method was used since one instrument were available for each tissue. FDR correction was applied using the Benjamini-Hochberg method [40].

##### MR analysis of *FADS1* and *FADS2* expression in brain single cell on cognitive function

In cell type-specific MR analysis, the putative causal effects of *FADS1* expression in two brain cell types and *FADS2* expression in one brain cell type on cognitive function were estimated by using the Wald ratio method [36]. FDR was computed using the Benjamini-Hochberg method [40].

##### MR analysis of *FADS1* and *FADS2* expression in whole blood on cognitive function

We further used three cis-eQTLs of *FADS1* expression and five cis-eQTLs of *FADS2* expression derived from whole blood respectively to estimate the causal effects of expression of these two genes on cognitive function by using IVW method. Moreover, a novel MR method with automated instrument determination (MRAID) was applied [41].

#### MR sensitivity analysis

We conducted a set of sensitivity analyses to estimate the effects using methods that were robust to other forms of pleiotropy using MR-Egger, weighted median, simple mode, and weighted mode, as each method can obtain consistent estimate of the causal effect if the pleiotropic effect is independent of the effect on the exposure. Cochrane’s Q test for inverse variance weighted analysis was conducted to assess the presence of heterogeneity between individual SNP [42].

#### Genetic colocalization analysis

To examine the posterior probability for a shared causal variant between *FADS1*/*FADS2* expression and cognitive function for the candidate MR signal [43], we used a Bayesian colocalization method that is noted as COLOC [30]. A colocalization probability (PP.H4) > 70% would suggest that the two genetic association signals are likely to share the same causal variant. Besides, we used an approximate colocalization analysis which is called LD check [44]. We estimated the linkage disequilibrium (LD) r^2^ between each cis-eQTL against all variants with GWAS *P* < 1 × 10^−3^ in the region associated with cognitive function. In this analysis, r^2^ > 0.7 between each cis-eQTL and cognitive function variants was considered as approximate colocalization.

## Results

We selected 45 omega-3 fatty acids variants as instruments, which were selected from Borges CM (*N* = 114,999). Besides, we selected 10 cis-eQTLs and nine cis-eQTLs respectively which is the strongest cis-eQTL for each brain tissue from the GTEx v8 database, and three cis-eQTLs from the MetaBrain data for tissue-specific MR analysis. For brain cell type-specific MR analysis, we used two cis-eQTLs from two cell types and one cis-eQTL from one cell type respectively that published from Bryois (*N* = 373). For instruments of *FADS1* and *FADS2* expression in whole blood, we selected three cis-eQTLs and five cis-eQTLs respectively that from eQTLGen Consortium (*N* ≤ 31,684). All the above cis-eQTLs were tested for Steiger filtering method so that there is no potential reverse causality. For *FADS1* and *FADS2* expression in different tissues, mean *F* statistics ranged from 8.5 to 581.5, indicating that most instruments were unlikely to be subject to weak instrument bias. *F* statistics for hippocampus and substantia nigra is less than 10 (Supplementary Table 2A). For *FADS1* and *FADS2* expression in different cell types, the *F* statistics only for inhibitory neurons is less than 10 (Supplementary Table 2B). We kept all instruments but with caution that three of these cis-eQTL dataset could suffer from weak instrument bias.

### Effect of omega-3 fatty acids on cognitive function

**Fig. 2:**
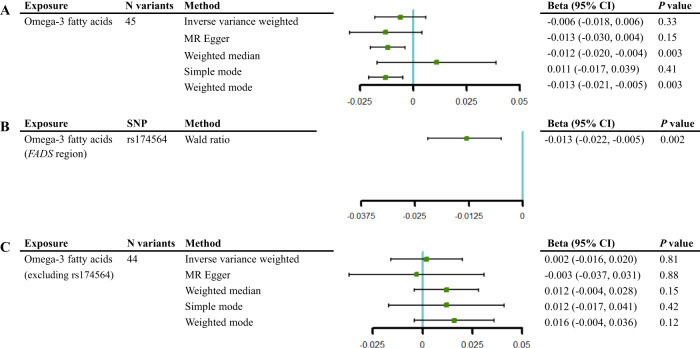
Mendelian randomization analysis of the causal effect of omega-3 fatty acids on cognitive function within and outside the *FADS* region. **A** All instruments of omega-3 fatty acids on cognitive function. **B** Single SNP within the *FADS* region of omega-3 fatty acids on cognitive function. **C** SNPs outside the *FADS* region of omega-3 fatty acids on cognitive function. The vertical line in this plot indicates the null of beta = 0 and the error bars correspond to 95% confidence intervals. CI confidence interval.

We investigated the causal effect of omega-3 fatty acids on cognitive function using genetic variants within and outside the *FADS* region. Little evidence was observed to support a causal effect using the IVW method [β = −6 × 10^−3^, 95% confidence interval (CI) (−1.8 × 10^−2^, 6 × 10^−3^), *P* = 3.3 × 10^−1^], although weighted median and weighted mode estimates suggested potential causal effects [β = −1.2 × 10^−2^, 95% CI (−2 × 10^−2^, −4 × 10^−3^), *P* = 3 × 10^−3^; β = −1.3 × 10^−2^, 95% CI (−2.1 × 10^−2^, −5 × 10^−3^), *P* = 3 × 10^−3^, respectively] (Fig. 2A). Besides, strong evidence of heterogeneity was observed for the overall effect of omega-3 fatty acids on cognitive function (*P*-value of the Q test = 5.5 × 10^−17^) (Supplementary Table 4). Specially, leave-one-out analysis indicated that the potential effect on cognitive function was driven by a single variant, rs174564, within the *FADS* region [β = −1.3 × 10^−2^, 95% CI (−2.2 × 10^−2^, −5 × 10^−3^), *P* = 2 × 10^−3^] (Fig. 2B, Supplementary Fig. 1). The estimated effect using instruments outside the *FADS* region showed little evidence by using IVW and the other sensitivity MR methods (*P* > 0.05) (Fig. 2C). In addition, the LDSC results showed that there was no genome-wide genetic correlation between omega-3 fatty acids and cognitive function after controlling for sample overlap (intercept = −1.3 × 10^−2^, *P* = 0.36, Supplementary Table 5).

### Tissue- and cell type-specific effect of *FADS1* and *FADS2* expression on cognitive function

**Fig. 3:**
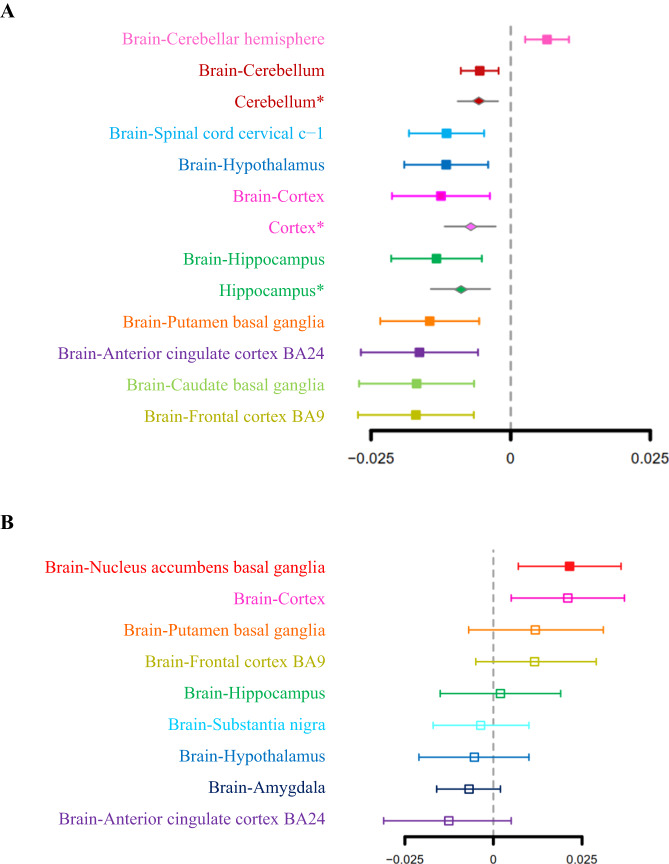
Forest plot illustrating the brain tissue-dependent association for *FADS1* and *FADS2* expression on cognitive function. **A** *FADS1*. **B** *FADS2*. The vertical line in this plot indicates the null of beta = 0 and the error bars correspond to 95% confidence intervals. Solid squares represented results that passed the LD check, while hollow squares represented results that failed the LD check. Asterisks represented results using the MetaBrain database.

**Fig. 4:**
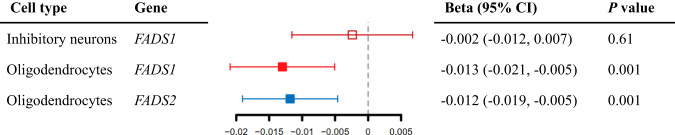
Forest plot illustrating the causal effect association for single cell gene expression of *FADS1* and *FADS2* on cognitive function. The vertical line in this plot indicates the null of beta = 0 and the error bars correspond to 95% confidence intervals. Solid squares represented results that passed the LD check, while hollow squares represented results that failed the LD check. CI confidence interval.

Due to the key role of *FADS* gene on cognitive function, we investigated the tissue- and cell type-specific causal effect of *FADS1* and *FADS2* expression on cognitive function (Figs. 3 and 4). As brain is closely related to cognitive function, we focused on explored the causal effect of *FADS1* and *FADS2* gene expression on cognitive function using cis-eQTL data from 10 and nine brain tissues respectively (e.g., amygdala, cortex, etc). The MR and colocalization analyses suggested putative causal effects of *FADS1* expression in 10 brain tissues and *FADS2* expression in one brain tissue on cognitive function, and these associations passed FDR threshold of 0.05: increased expression levels of *FADS1* gene in cerebellar hemisphere showed a cognitive function maintenance effect. While, decreased expression levels of *FADS1* in nine additional brain tissues showed effects on maintaining cognitive function, including cerebellum, spinal cord cervical c-1, hypothalamus, cortex, hippocampus, putamen basal ganglia, anterior cingulate cortex BA24, caudate basal ganglia, and frontal Cortex BA9. In addition, the significant results with colocalization evidence for cerebellum, cortex and hippocampus were validated in the MetaBrain data and were all directionally consistent with the MR effects in GTEx (Fig. 3A). For *FADS2*, increased expression levels in nucleus accumbens basal ganglia showed a possible maintenance effect of cognitive function. The MR and colocalization results suggested little evidence to support causality for *FADS2* in other eight brain tissues (Fig. 3B, Supplementary Table 6).

Secondly, we estimated the cell type-specific causal effect of gene expression of *FADS1* and *FADS2* on cognitive function using brain single-cell cis-eQTL data. *FADS1* and *FADS2* expression in one cell type showed MR and colocalization evidence: decreased levels of *FADS1* and *FADS2* expression in oligodendrocytes showed cognitive function maintenance effect. Causal effect of *FADS1* expression on cognitive function was not observed in inhibitory neurons (Fig. 4, Supplementary Table 7).

**Fig. 5:**
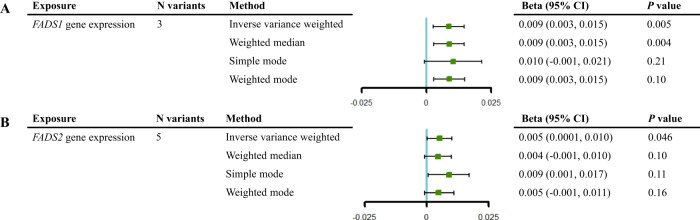
Mendelian randomization analysis of the causal effect of *FADS1* and *FADS2* gene expression in whole blood on cognitive function. **A** *FADS1*. **B** *FADS2*. The vertical line in this plot indicates the null of beta = 0 and the error bars correspond to 95% confidence intervals. CI confidence interval.

**Fig. 6:**
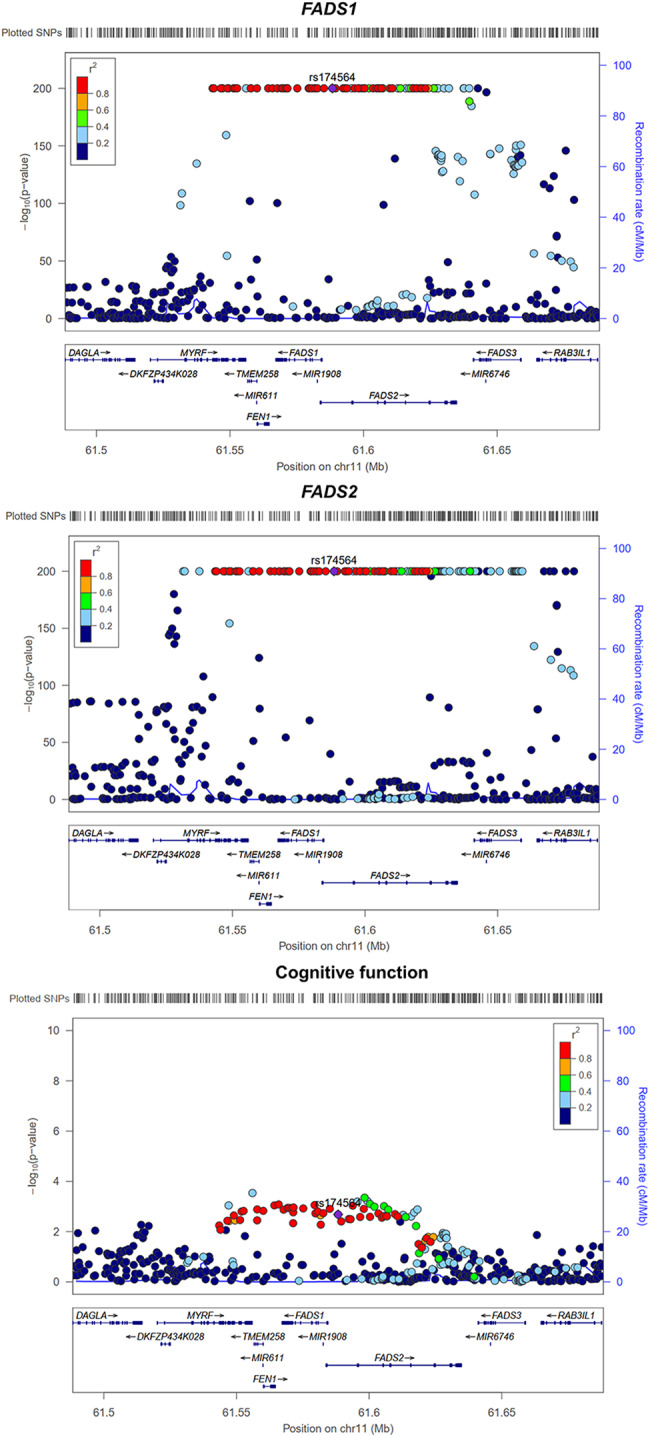
Regional association plots of *FADS1* and *FADS2* expression in whole blood on cognitive function in the *FADS* region. The candidate signal within *FADS* region is rs174564.

In order to further verified the role of *FADS1* and *FADS2* gene expression on cognitive function in whole blood, we estimated the causal effect using three cis-eQTLs for *FADS1* and five cis-eQTLs for *FADS2* respectively. MR analysis indicated that increased expression levels of *FADS1* and *FADS2* in whole blood showed effects on cognitive function maintenance [IVW β = 9 × 10^−3^, 95% CI (3 × 10^−3^, 1.5 × 10^−2^), *P* = 5 × 10^−3^; IVW β = 5 × 10^−3^, 95% CI (1 × 10^−4^, 1 × 10^−2^), *P* = 4.6 × 10^−2^; respectively]. In sensitivity analysis, weighted median suggested that increased expression levels of *FADS1* was associated with maintenance of cognitive function [β = 9 × 10^−3^, 95% CI (3 × 10^−3^, 1.5 × 10^−2^), *P* = 4 × 10^−3^], while the estimates showed little causal evidence using other sensitivity MR methods (Fig. 5). Little evidence of heterogeneity was observed (*P*-value of all the Q test > 0.05) (Supplementary Table 4). The MRAID method showed that *FADS1* expression in whole blood had robust causal effect on cognitive function (*P* = 0.02) and directionally consistent with the MR effects from the IVW method, while the causal effect of *FADS2* were not observed (Supplementary Table 8). After performing colocalization analysis with the candidate MR signal, we observed compelling evidence of gene colocalization between expression of *FADS1* and *FADS2* and cognitive function (PP.H4 = 86.6% and 88.4%, respectively) (Fig. 6, Supplementary Table 9).

Furthermore, to understand the link between omega-3 fatty acids variants and *FADS* gene variants, we estimated the LD between them. The omega-3 fatty acids variant rs174564 is located in the intron of *FADS2* gene and it is in strong LD (r^2^ > 0.7) with several of the *FADS1* and *FADS2* cis-eQTLs/instruments we used. This suggested that omega-3 fatty acids and *FADS1/FADS2* cis-eQTLs are likely to represent the same genetic signal in the *FADS* region. Therefore, the effect of *FADS1/FADS2* expression on cognitive function could be related to the omega-3 fatty acids variant rs174564. The pairwise LD r^2^ between each cis-eQTL and rs174564 was presented in Supplementary Table 10.

Finally, we attempted to identify potential mechanistic pathways between omega-3 fatty acids, *FADS1/FADS2* genes, and cognitive function through MELODI Presto [45]. The results showed that potential intermediates between omega-3 fatty acids or *FADS1/FADS2* genes and cognitive function were mostly associated with metabolic or neurological diseases, such as non-insulin dependent diabetes, metabolic syndrome, obesity and Alzheimer’s disease (Supplementary Table 11).

## Discussion

In this study, we found that *FADS1* and *FADS2* expression in different brain tissues and cell types showed causal effect on cognitive function using genetic tools. Data on the expression of *FADS1* and *FADS2* in whole blood further confirmed this finding. Our results revealed that *FADS1* and *FADS2* are likely to be two causal genes influencing cognitive function, while the *FADS* gene as potential target gene, may be functional especially in specific cell type.

Previous epidemiology studies reported a protective effect of omega-3 fatty acids on cognitive function, and this effect is particularly pronounced in individuals with early and mild cognitive impairment [46]. However, no benefit was observed when subjects with diagnosed Alzheimer’s disease were supplemented with omega-3 fatty acids as well as in many other population-based studies [47–49]. These inconsistencies may be attributed to interventions in RCTs that have been carried out too late to against the progression of cognitive impairment and are vulnerable to confounding factors. In our MR analysis, we found a weak negative association between omega-3 fatty acids and cognitive function using some MR methods, while the sensitivity analysis suggested strong evidence of heterogeneity, which suggested that a few genes may drive the causal effect between the two. Leave-one-out analysis further suggested that the effect of omega-3 fatty acids on cognitive function is more likely to be driven by SNP within the *FADS* gene region rather than a general effect of omega-3 fatty acids.

As a natural extension, we investigated the impact of *FADS1* and *FADS2* gene expression on cognitive function. The *FADS* variants have been reported to be associated with cognitive function in previous studies, but the association has only been studied in the context of the effect of *FADS* gene variation on children or offspring [50–53]. Genetic variants in the *FADS1/FADS2* region are associated with maternal long-chain polyunsaturated fatty acid status and could modified cognitive development of infants [50]. In addition, *FADS1/FADS2* genetic variants have been reported to be associated with behavioral outcomes in children [52, 53]. However, one issue to be resolved is whether there is an association between *FADS* gene expression and cognitive function, and whether this association is influenced by tissue type, especially brain tissue. Our tissue-specific MR analysis showed that increased levels of *FADS1* expression in cerebellar hemisphere and *FADS2* expression in nucleus accumbens basal ganglia may maintain cognitive function, while decreased levels of *FADS1* expression in other nine brain tissues, including cerebellum, spinal cord cervical c-1, hypothalamus, cortex, hippocampus, putamen basal ganglia, anterior cingulate cortex BA24, caudate basal ganglia, and frontal Cortex BA9, may benefit cognitive function maintenance. It is accepted that the cerebellum played a possible role in the mediation of cognitive processes [54]. Previous studies have showed that individuals with Parkinson’s disease had significant atrophy of left cerebellar hemisphere [55, 56]. A broad variety of cognitive and linguistic deficits can occur after cerebellar damage [57–59]. The most popular mechanism of cerebellar involvement in cognitive functions is Schmahmann’s dysmetria of thought theory, which assuming that the way the cerebellum regulates movement may also influence mental processes [60]. Besides, basal ganglia are critical for several cognitive, motor and emotional functions and are part of a complex functional circuit [61–63]. Early animal experiments confirmed the relationship between basal ganglia and cognitive and memory function, which pointed out that this relationship may be related to the cholinergic neuronal impulse transmission in the basal ganglia and the role of dopamine neurons for reward learning [64, 65]. Human studies have also reported that basal ganglia may play an integrative role in cognitive information processing and that the electrical activity of multifunctional clusters of neuronal populations may underlie this nonspecific integrative effect [66]. In this study, we revealed a putative causal mechanism that increased expression levels of *FADS1* gene in cerebellar hemisphere and *FADS2* gene in nucleus accumbens basal ganglia are associated with maintenance of cognitive function.

It is important to notice that eQTL effect of the same gene could be different dependent on the tissues or cell types of the human brain. In tissue-specific MR analysis, we observed that both *FADS1* and *FADS2* expression in cortex showed MR evidence, which decreased *FADS1* expression levels and increased *FADS2* expression levels showed maintenance effect on cognitive function. However, the causal effect of *FADS2* were not confirmed by colocalization evidence. Similar with cortex, decreased expression levels of *FADS1* in anterior cingulate cortex BA24 and frontal cortex BA9 was associated with maintaining cognitive function. This directional inconsistency may be due to the limitation of tissue sample size or there may be different pathways involved in *FADS1*/*FADS2* expression in cortex on cognition. More datasets of larger independent tissue-specific eQTL data and additional genetic methods, such as transcriptome-wide association study (TWAS), should be considered in future studies to further improve the statistical power and identify true causal genes with functions [67–70]. Previous studies have affirmed the role of the anterior cingulate cortex and frontal cortex in social cognition and cognitive control [55, 56, 71, 72], and we supplied new evidence for this association at genetic level. Furthermore, our cell type-specific MR analysis used single-cell brain cis-eQTL data highlighted the important role of *FADS1/FADS2* gene in oligodendrocytes. Recently, Kenigsbuch et al. [73] confirmed that oligodendrocyte state was associated with brain pathologies among multiple central nervous system diseases. Our findings provided new evidence that decreased expression levels of *FADS1/FADS2* in oligodendrocytes could influence cognitive function. The potential mechanism causing the differences between tissues and cells need further investigation.

To further verify our findings, we also used cis-eQTL data in whole blood and found a protective effect of *FADS1* and *FADS2* expression in blood on cognition. Additionally, the causal effect of *FADS1* gene was also confirmed by MRAID method, which provided additional evidence to prove the robustness of this finding. As one of the main MR approaches, the IVW method relies on pre-selected independent SNPs as instruments for MR analysis and could not account for horizontal pleiotropy [74]. MRAID uses multiple correlated genetic variants and account for correlated and uncorrelated pleiotropy [41]. While, the causal effect of *FADS2* gene was only observed using the IVW method. Despite the good statistical power from both IVW or MRAID analysis [41], the effect of *FADS2* on cognitive function needs to be investigated in future studies. Importantly, our colocalization evidence confirmed the causal effect of *FADS1/FADS2* expression levels on cognitive function. It is well known that *FADS1* and *FADS2* polymorphisms could modulate fatty acid metabolism [75]. The results from MELODI Presto also identified and prioritized metabolic and neurological diseases as potential intermediates between omega-3 fatty acids or *FADS1/FADS2* genes and cognitive function, which provided direction for future mechanistic studies.

There are some strengths in our study. First, we have used large-scale GWAS data of omega-3 fatty acids, tissue and single-cell sequencing cis-eQTL data of gene expression and GWAS data of cognitive function, which brought good instrument strength and statistical power to our study. Second, traditional studies tend to focus on the association between omega-3 fatty acids and cognition, while we proposed for the first time that *FADS1* and *FADS2* expression in multiple brain tissues and cell types had different effect on cognitive function. Third, we have supplemented the mechanism of cognition at the genetic level by providing evidence to prioritize *FADS1* and *FADS2* as two potential target genes on cognition, which could be functional in brain.

Our study has several limitations. Firstly, the instruments of omega-3 fatty acids and a small proportion of the outcome samples were obtained from the UK Biobank, which have minor sample overlap issue. However, there was no sample overlap between cis-eQTL data and the outcome GWAS, which means the vast majority of the MR results will not be influenced by the sample overlap issue. Secondly, there were limited number of instruments for the cis-eQTL data, which means most of the MR sensitivity methods such as MR-Egger were not applicable. However, we systematically conducted colocalization analysis to enhance the causal evidence of our findings. Thirdly, the *F* statistics of hippocampus, substantia nigra and inhibitory neurons were lower than the common threshold of 10, the weak instrument bias need to be carefully considered when interpreting the findings. However, our top findings were observed in oligodendrocytes, which showed good instrument strength. Large-scale single-cell eQTL studies are needed in the future to provide better statistical power.

In conclusion, our MR analysis showed novel insight between *FADS1/FADS2* gene expression and cognitive function by using tissue and single cell cis-eQTL data and state-of-the-art methods such as genetic colocalization. Integrating these novel data and methods suggested that *FADS1* and *FADS2* expression levels could influence cognitive function in different brain tissues and cell types. Our results provided clues for the understanding of the genetic mechanism of cognitive function and improved the current knowledge of *FADS* gene and cognition. Future studies are needed to prioritize *FADS1*/*FADS2* as potential target genes for maintenance of cognitive function.

### Supplementary information

Supplementary Table 1-11

Supplementary Figure

## Data Availability

GWAS data of omega-3 fatty acids are available from the UK Biobank (https://www.ukbiobank.ac.uk). Brain tissue cis-eQTL data of *FADS* gene can be obtained from the GTEx project (v8; https://gtexportal.org/home) and the MetaBrain consortium (https://www.metabrain.nl). Single-cell sequencing cis-eQTL data of *FADS* gene can be accessed from the respective publication. The cis-eQTL data of *FADS* gene expression in whole blood are available on the eQTLGen Consortium (https://eqtlgen.org). The GWAS summary statistics for cognitive function are available from the respective publication.

## Code availability

The main statistical analyses were conducted using TwoSampleMR R package (v.0.5.6). Colocalization analysis was conducted using coloc R package (v.5.2.0).
