# Supplementary material for: Brain tissue- and cell type-specific eQTL Mendelian randomization reveals efficacy of FADS1 and FADS2 on cognitive function
Source: Transl Psychiatry. 2024 Feb 5;14:77. doi: 10.1038/s41398-024-02784-4 (PMC10844634; doi:10.1038/s41398-024-02784-4)
Supplement: Supplementary file 2 — Supplementary Figure [file 41398_2024_2784_MOESM2_ESM.docx]

**Supplementary Figure 1**


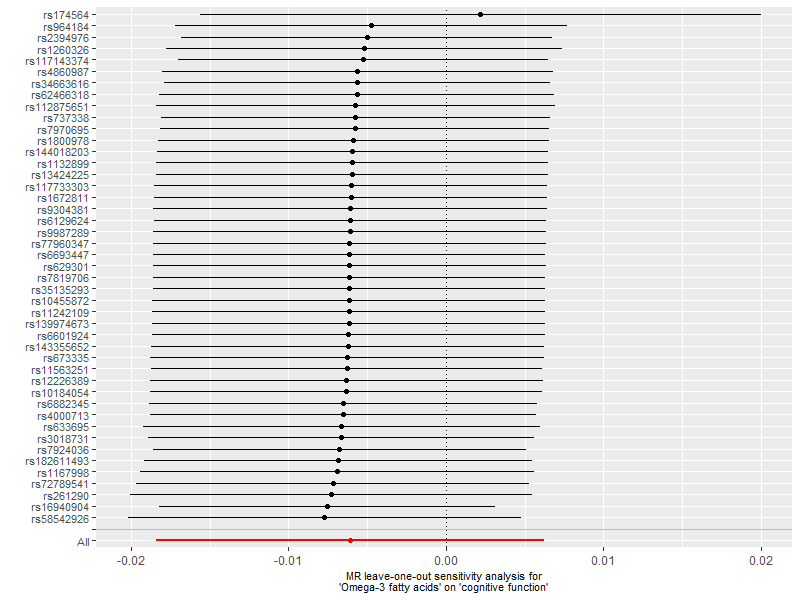


Supplementary Figure 1. Leave-one-out analysis for omega-3 fatty acids on cognitive function
